# Os-Artificial

**Published:** 1868-08

**Authors:** Charles L. Houghton

**Affiliations:** Poughkeepsie, N. Y.


### OS-ARTIFICIAL.

BY DR. CHARLES L. HOUGHTON.

This material, frequently called "Oxy Chloride of Zinc," "bone filling," etc., was first introduced to the profession in the fall of 1859, by Dr. Charles H. Roberts.

It has of late attracted the attention of leading members of the profession as a material for filling over sensitive dentine and exposed pulps, with what success the profession can judge from reports of those making it their mode of practice.

It has been my practice, for the last eight years, to fill all cases of slight nerve exposure boldly with the os-artificial, having no fear of any bad after effect, and out of ninety-seven cases treated in this manner, during the two years I kept careful record, only three cases proved failures, which, as shown by an examination after extracting, was caused by carelessness in pressing the filling too hard on the exposed pulp.

I have, for several years, strongly advocated the use of os-artificial as a material for filling over and protecting exposed nerves, *knowing*, from experience, that the operation, if performed carefully, would prove successful, and in 1864 wrote an article on the subject for one of the Dental journals, which, however, was returned with a long letter from the editor expressing his belief that it was an impossibility to treat a tooth in that manner, and the pulp retain its vitality.

The os-artificial is the most perfect non-conductor in use, and as a material for filling in the bottoms of large sensitive cavities, and finishing with gold or tin, as described in my article on the subject in the December number of the REGISTER, has proved highly satisfactory in every case. I have used it in this connection ever since its introduction, ten years ago.

The escharotic properties of the liquid frequently causes severe pain at first, which, however, passes off in a few moments. This painfulness can be obviated in a degree by introducing the filling while in a stiff paste, and not while the surface looks watery.

*Poughkeepsie, N. Y.*

